# The Cardiovascular Effects of GLP-1 Receptor Agonists

**DOI:** 10.1111/j.1755-5922.2010.00256.x

**Published:** 2010-12-19

**Authors:** Theodore Okerson, Robert J Chilton

**Affiliations:** 1 Diabetes Research, Amylin PharmaceuticalsSan Diego, CA, USA; 2 Department of Medicine, University of TexasSan Antonio, TX, USA

**Keywords:** Cardiovascular, Diabetes, Exenatide, GLP-1, Hypertension

## Abstract

Glucagon-like peptide-1 receptor (GLP-1R) agonists have been shown to regulate blood glucose concentrations by mechanisms including enhanced insulin synthesis/secretion, suppressed glucagon secretion, slowed gastric emptying, and enhanced satiety. GLP-1 receptors have also been identified in the heart, kidneys, and blood vessels, leading to the hypothesis that GLP-1R agonists may affect cardiovascular function or cardiovascular disease (CVD). The aim of this literature review was to assemble and assess preclinical and clinical data of potential medical importance regarding the cardiovascular effects of GLP-1R agonists. Preclinical studies with the GLP-1R agonists GLP-1, exenatide, or liraglutide provided evidence that GLP-1R stimulation favorably affects endothelial function, sodium excretion, recovery from ischemic injury, and myocardial function in animals. Similar observations have been made in exploratory studies on GLP-1 infusion in normal subjects and patients with type 2 diabetes. *Post hoc* analyses of phase III studies of patients with type 2 diabetes treated with exenatide(bid or qw) or liraglutide(qd) showed that these GLP-1R agonists reduced blood pressure, an effect largely independent of weight loss, and that liraglutide slightly increased heart rate. Preliminary data also indicated that GLP-1R agonists reduced markers of CVD risk such as C-reactive protein and plasminogen activator inhibitor-1. Ongoing studies are examining the effects of administering GLP-1R agonists to patients at risk of CVD, postangioplasty patients, post-CABG patients, and patients with heart failure. Additional studies should provide meaningful data to determine whether GLP-1R agonists provide unique treatment benefits to patients at risk for or with established CVD.

## Introduction

Glucagon-like peptide-1 receptor (GLP-1R) agonists have been investigated for treating type 2 diabetes mellitus (T2DM) since the early 1990s because of their ability to enhance glucose-dependent insulin secretion. The cessation of GLP-1-stimulated insulin release when blood glucose concentrations are <55 mg/dL is probably responsible for the low incidence of severe hypoglycemia observed in phase III clinical trials of the GLP-1R agonists exenatide and liraglutide [1]. Uniquely, GLP-1R agonists exert coordinated effects on mechanisms of glucose release and nutrient uptake, suppress inappropriately elevated glucagon secretion, slow gastric emptying, and increase satiety with the net result of reduced body weight [2]. Weight loss and lower blood glucose concentrations may reduce patients’ risk for cardiovascular disease (CVD) [3–5].

In addition, GLP-1R agonists are hypothesized to have pleiotropic effects on the cardiovascular system. This is a particularly important research area for patients with T2DM since these individuals are at increased risk for CVD and recover from cardiovascular events less well than patients without diabetes [6–8]. Type 2 diabetes is frequently associated with proatherogenic risk factors including hypertension, overweight or obesity, and dyslipidemia with elevated proinflammatory markers and procoagulant factors [9]. However, patients with T2DM also demonstrate endothelial dysfunction and impaired vasodilatation [9], microvascular disease (particularly in the myocardial microcirculation [10–11]), increased arterial stiffness [12], left ventricular hypertrophy [13], and cardiac fibrosis [14]. To improve the cardiovascular outcomes of patients with T2DM, comprehensive risk reduction was recommended by a joint committee of the American Heart Association and the American Diabetes Association, specifically to achieve an A1C <7% or as close to normal (<6.0%) as possible without significant hypoglycemia, weight loss to reduce multiple CVD risk factors, aggressive management of hypertension, treatment of dyslipidemia, and therapy to prevent platelet aggregation [15].

The purpose of this review is to summarize published preclinical and clinical studies on the cardiovascular effects of GLP-1R agonists and to assess the evidence as a whole. Search terms included “glucagon-like peptide,”“GLP,”“exenatide,” and “liraglutide” and papers published prior to October 1, 2009 were selected based on their relevance to CVD, cardiovascular risk, or hypertension.

## GLP-1 Receptor Agonists

Glucagon-like peptide-1 (GLP-1) is a peptide hormone primarily synthesized in the distal ileum and colon and released into the circulation in response to food intake. GLP-1(7–36)NH_2_ is the primary active isoform, although it is degraded with a half-life of 2 min to GLP-1(9–36) by dipeptidyl peptidase-4 (DPP-4) [16]. In patients with T2DM, the postprandial GLP-1 secretory response to a meal is significantly diminished and it is hypothesized that this contributes to the pathogenesis of diabetes [17–18].

Discovery of the pleiotrophic effects of GLP-1 on blood glucose control led to the development of GLP-1R agonists with extended half-lives for the treatment of diabetes. The first GLP-1R agonist (incretin mimetic) approved for clinical use was exenatide (exendin-4), which is administered by subcutaneous injection. Discovered in 1990, this naturally occurring 39-amino-acid peptide was isolated from the saliva of the Gila monster (Heloderma suspectum). It shares 53% homology with mammalian GLP-1 and stimulates GLP-1R, yet is not degraded by DPP-4. Exenatide's circulating half-life is 60–90 min, which is sufficient for twice daily (bid) administration [2]. A once-weekly formulation (exenatide QW) has been submitted for regulatory review to the US FDA.

Liraglutide, a derivative of GLP-1 administered once daily (qd), has also been approved for clinical use in the United States and Europe. Liraglutide differs from native GLP-1 by the substitution of arginine 34 for lysine. However, the lysine at position 26 is acylated with a fatty acid [-CO-(CH_2_)_14_ CH_3_] that facilitates binding to serum albumin, creating a liraglutide: albumin complex extending the half-life of liraglutide sufficiently for qd dosing. The addition of a fatty acid and formation of a complex with albumin are key molecular differences between liraglutide and GLP-1 [19–20]. The metabolites of liraglutide differ from those of exenatide due to liraglutide's susceptibility to cleavage by DPP-4.

The majority of studies on the cardiovascular effects of GLP-1R agonists have been completed with native GLP-1, exenatide, or liraglutide. Additional GLP-1R agonists in clinical trials include lixisenatide (AVE0100), taspoglutide, and albiglutide. Because GLP-1R agonists in development for clinical applications are resistant to cleavage by DPP-4, studies on the physiological effects of GLP-1 metabolites will not be addressed.

## The GLP-1 Receptor

**Figure 1:**
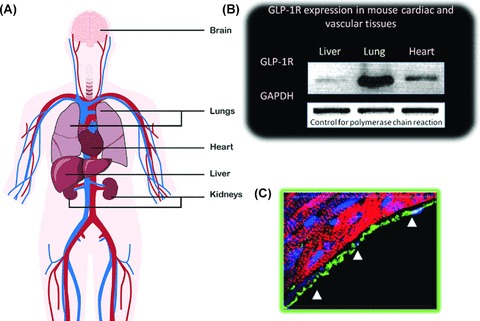
Nongastrointestinal GLP-1R. (A) Overview of organs and tissues with GLP-1R. (B) Quantitation of GLP-1R expression in mouse tissues. (C) Immunohistochemical staining (green) demonstrating localization of GLP-1R in the endocardium (B, C reproduced with permission from [22]).

The human GLP-1R is a G-protein coupled receptor consisting of 463 amino acids including a large extracellular domain and seven transmembrane domains [21]. GLP-1R expression has been identified in pancreatic islet cells, lung, brain, stomach, kidney, liver, and heart in animal studies [21–22]. Recently, GLP-1R expression has been further localized in mice to cardiomyocytes, the endocardium, microvascular endothelium, and medial aortic smooth muscle cells [22] (Figure 1).

Multiple intracellular mediators for GLP-1R signaling have been identified in different tissues. It is generally agreed that the GLP-1R signals via the stimulatory G-protein Gs to increase cyclic AMP accumulation and activate protein kinase A. GLP-1R stimulation also increases intracellular calcium concentrations and activates calcium-dependent signaling cascades in pancreatic beta cells [21]. Activation of the phosphatidylinositol-3-kinase and mitogen-activated protein kinase pathways has been observed in insulin-secreting cell lines [23]. When GLP-1 was infused into isolated mouse hearts, cGMP production markedly increased compared with nontreated hearts, suggesting a role for this signaling pathway in GLP-1 activity [22]. If the signaling mechanisms observed in pancreatic cells are stimulated by GLP-1 in vascular and cardiac cells, vascular tone, muscle contractility, cell growth/differentiation, extracellular matrix remodeling, and apoptosis may be affected.

In humans, GLP-1R activity depends on GLP-1R agonist concentration [24–25]. This relationship may explain the observation that the DPP-4 inhibitor sitagliptin has lesser effects on postprandial glucose than exenatide, as sitagliptin yields a lower dose of GLP-1R agonist [26].

## Preclinical Studies on the Cardiovascular Effects of GLP-1 Receptor Agonists

**Figure 2:**
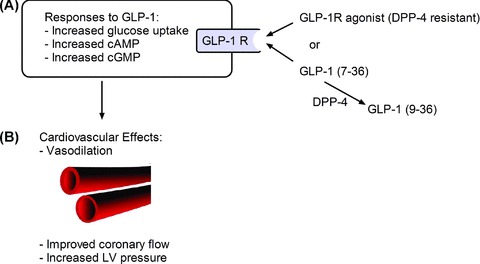
Schematic of GLP-1R signaling in heart cells. (A) Intracellular signaling. (B) Vascular consequences of GLP-1R signaling [22].

Numerous preclinical studies in rodents have investigated cardiovascular mechanisms of GLP-1R stimulation. Observed responses have included sympathetic nervous system activation, reduced triglyceride absorption, increased natriuresis, vasorelaxation, enhanced NO production, increased cardiac glucose uptake, and augmented cardiac contractility (Figure 2; Table 1).

**Table 1:** Physiological effects of GLP-1R agonists in animal studies

Topic	Research system	Results of GLP-1R agonist	Reference
Nervous system	Rats: exenatide or GLP-1	↑BP and ↑HR (central and peripheral administration)	[28]
	Rats: exenatide	↑HR, regionally selective effects on vascular conductance. β-adrenergic stimulation contributed to vasodilator and cardiac effects	[29]
Intestines	Rats: GLP-1	↓Triglyceride absorption and ↓apolipoprotein production	[30]
Kidneys	Dahl salt-sensitive rats: GLP-1	Less hypertension, ↑urine flow, ↑Na^+^ excretion, ↓proteinuria with ↑Na^+^ diet	[31]
	Salt-sensitive obese (db/db) salt-loaded mice: exenatide	Less hypertension, ↑Na^+^ excretion, attenuated AngII-induced high-salt sensitivity	[32]
	Corticosterone-treated rats: exenatide	Normalized BP independently of body weight	[33]
Blood vessels	Rat mesenteric postcapillary venules: GLP-1	↓Microvascular permeability after exposure to LPS	[34]
	Rat thoracic aorta: GLP-1, exenatide, DMB	↑Vasorelaxation	[35]
	Rat thoracic aorta: GLP-1, exenatide	↑Vasorelaxation	[36]
	Rat pulmonary circulation: GLP-1	↑Vasorelaxation	[37]
	Rat femoral artery: exenatide	↑Vasorelaxation	[38]
Heart	Isolated rat heart: GLP-1	↑Glucose uptake, ↑NO production, ↑coronary flow	[39]
	Isolated mouse heart: GLP-1, exenatide	↑LVDP, ↑Coronary flow, ↑ glucose uptake	[22]

Na^+^, sodium ion; AngII, angiotensin II; BP, blood pressure; DMB, 6,7-dichloro-2-methylfulfonyl-3-N-tert-butylamino-quinoxaline; GLP-1R, glucagon-like peptide-1 receptor; HR, heart rate; LPS, lipopolysaccharide; LVDP, left-ventricular end diastolic pressure; NO, nitric oxide.

A recent study in mice has also suggested that exenatide reduces macrophage adhesion to the endothelium, the key initiating step in formation of atherosclerotic plaques [27]. The majority of these findings have been observed in >1 model system by multiple research groups, suggesting that the results of GLP-1R stimulation are reproducible. Observation of responses to GLP-1R agonists in isolated blood vessels and hearts suggests that GLP-1 agonists act directly on these target organs and not by modulating insulin release or systemic glucose concentrations, which are controlled in these experiments. The effects of GLP-1R agonists have also been studied in models of MI (ischemia with or without reperfusion) and heart failure (HF). While it is difficult to compare studies conducted with methodological differences, improvement in postischemia cardiac function or infarct size with GLP-1 or exenatide treatment was observed in seven of the nine studies identified (Table 2). Interestingly, improved cardiac function or reduced infarct size was observed with mild (e.g., low-flow or short-term) ischemia as well with permanent vessel occlusion and some effects were observed in isolated hearts as well as in intact animals (Table 2). The most striking results were observed in *in vivo* studies with longer follow-up times, particularly the Timmers study in pigs (3 days) [40] and the Noyan–Ashraf study in mice (28 days) [41]. In the Timmers study, compared to saline treatment, exenatide significantly reduced infarct area (32.7 ± 6.4 vs. 53.6 ± 3.9%, *P*= 0.031, respectively) and myocardial stiffness (0.12 ± 0.06 mm Hg/mL vs. 0.22±0.07 mm Hg/mL, *P*= 0.004). In addition, systolic wall thickness (47.3 ± 6.3% vs. 8.1 ± 1.9%, *P* < 0.001) and dPdt_m_ (1611 ± 91 mm Hg/s vs. 1345 ± 75 mm Hg/s, *P* < 0.05) increased with exenatide treatment [40]. In the Noyan–Ashraf study, treatment with liraglutide versus saline produced marked differences in outcome, respectively: cardiac rupture occurred in fewer mice (20% vs. 77%, *P*= 0.0001); infarct size was reduced (21 ± 2% vs. 29 ± 3%, *P*= 0.02); and cardiac output improved (12.4 ± 0.6 mL/min vs. 9.7 ± 0.6 mL/min, *P*= 0.02). In contrast, the negative studies examined infarct size in pigs after only 2 or 2.5 h of reperfusion, respectively [42–43].

**Table 2:** Effects of GLP-1R agonists in animal models of ischemic injury

Research System	Injury protocol	Results of GLP-1R agonist	Reference
Isolated rat heart: GLP-1 before ischemia and during reperfusion.	30 min of low-flow ischemia, 30-min reperfusion	↑Glucose uptake, ↑LV function	[39]
Isolated mouse heart: GLP-1, GLP-1(9–36) or exenatide before ischemia and during reperfusion	30 min of no-flow ischemia, 40-min reperfusion	↑Developed pressure, ↑coronary flow with all 3 treatments	[22]
Isolated rat heart: exenatide, GLP-1(9–36) during reperfusion	45 min of no-flow ischemia followed by reperfusion for 120 min	Ischemic area decreased >2-fold (*P* < 0.05) for exenatide versus control. GLP-1(9–36) had no effect	[44]
Rat: GLP-1+DPP-4 inhibitor infused continuously beginning prior to ischemia	Left anterior coronary artery ligated for 30 min. Reperfusion for 120 min.	Area of ischemic injury reduced >2-fold (*P* < 0.001) versus saline or DPP-4 inhibitor alone.	[45]
Pig: liraglutide pretreatment for 3d	LAD ligation for 40 min followed by 2.5-h reperfusion	Infarct size did not differ significantly. ↑HR with liraglutide.	[42]
Pig: continuous GLP-1 beginning prior to ischemia	1 or 2 diagonal arteries of the LAD sutured for 60 min followed by 2-h reperfusion	No difference in infarct size. ↓Interstitial pyruvate and lactate.	[43]
Dog: continuous infusion of GLP-1	10 min of balloon occlusion of LCx followed by 24-h reperfusion	Regional wall motion recovered earlier and more completely than controls	[46]
Pig: exenatide prior to reperfusion	LCx occluded for 75 min followed by reperfusion for 3 days	↓Infarct size (*P*= 0.03) and preserved cardiac function. ↓Apoptosis	[40]
Mouse: liraglutide pretreatment for 7 days	LAD ligation. Biochemical and histological analyses at 4 days, survival analyses at 28 days	Reduced infarct size, less rupture, improved survival. Cardioprotective genes activated. MMP-9 activity reduced	[41]

DPP, dipeptidyl peptidase; HR, heart rate; LAD, left anterior descending coronary artery; LCx, left circumflex coronary artery; MMP, matrix metalloprotease.

GLP-1R stimulation has also been observed to improve cardiac function in animal models of HF. In a pacing-induced dilated cardiomyopathy canine model induced over 28 days and then treated for 2 days, an infusion of GLP-1 (7–36) increased myocardial glucose uptake versus saline (7.9 ± 0.5 μmol/min vs. 4.7 ± 0.3 μmol/min, *P* < 0.05) and improved LV ejection fraction (EF) over time (from 28 ± 1% to 38 ± 5%) [47]. The longest duration animal study to date investigated effects of continuous infusion of GLP-1 over 3 months in 9-month old spontaneously hypertensive rats prone to HF. In these animals, GLP-1 infusion versus saline infusion increased survival at 12 months (72 vs. 42%, *P*= 0.008), increased cardiac stroke volume (1.4 ± 0.1 mL vs. 1.0 ± 0.1 mL, *P*= 0.001), increased cardiac output (543 ± 22 mL/min vs. 402 ± 43 mL/min, *P*= 0.001) and improved LVEF (82 ± 3% vs. 70 ± 4, *P*= 0.016) [48].

Although questions remain as to the role of the endothelium in GLP-1 stimulated vasodilatation and which of the preclinical models are most predictive of human response, these preclinical studies support a transition to human studies on the cardiovascular effects of GLP-1R agonists.

## Clinical Data on the Cardiovascular Effects of GLP-1 Receptor Agonists

Current clinical data on the cardiovascular effects of GLP-1R stimulation in humans have been provided by small, exploratory studies on intravenous administration of GLP-1 or through *post hoc* analyses of data obtained from phase III clinical trials of subcutaneously administered GLP-1R agonists. Short-term studies of direct effects of GLP-1R agonists on the sympathetic nervous system, renal excretion of sodium, and vasodilatation have been published (Table 3). Less effect of GLP-1R agonists on cardiac sympathetic nerve stimulation seems to have been observed in humans than in animals, but this may differ between GLP-1R agonists and between species. In the LEAD-6 study, a larger increase in heart rate was observed with liraglutide qd than with exenatide bid (3 vs. 1 bpm, respectively, *P*= 0.0012) [49]. Furthermore, the renal and vascular effects of GLP-1 in humans are qualitatively similar to those in animal studies. In contrast to the significant amount of preclinical data available, only one study has been published to date in patients with acute MI after successful reperfusion [57]. In this study, Dr. Nikolaidis and colleagues compared the in-hospital outcomes of a 72-h infusion of recombinant GLP-1 (1.5 pmol/kg/min) added to standard therapy with standard therapy alone in 21 high-risk patients with acute MI and LV dysfunction. In 10 patients, GLP-1 administration was initiated an average of 212 min after reperfusion and was associated with significantly improved LVEF (29–39% compared with no change in the control group, *P* < 0.01) and contractile function (−21% in regional wall motion score index vs. no change, *P* < 0.001) measured 6–12 h after infusion. In the small subset of patients (four per group) for whom 120-day data were available, the effect on LVEF persisted. Trends toward shorter hospital stays (6 vs. 10 days) and reduced in-hospital mortality (one death in the GLP-1 treated group vs. three in the control group) were also observed [57].

**Table 3:** Effects of GLP-1R agonists in human studies

Topic	Study design	Results of GLP-1R agonist	Reference
Nervous system	Continuous sc infusion of GLP-1 for 48 h in six patients with T2DM	No change in HR. No significant difference in BP	[50]
	IV infusion of GLP-1 for ∼1 h in seven healthy volunteers	Increased muscle sympathetic nerve activity but no effect on HR, BP, or cardiac neural activity	[51]
	Ph III study comparing exenatide bid and liraglutide qd in 464 patients over 26 weeks	HR increased from baseline +0.69 bpm for exenatide versus +3.28 bpm for liraglutide (*P*= 0.0012)	[49]
Kidneys	IV infusion of GLP-1 for 3 h in 15 normal and 16 obese subjects given salt bolus	↑Na^+^ in both groups. ↓H^+^ excretion and ↓glomerular hyperfiltration in obese subjects	[52]
	IV infusion of GLP-1 for 3 h in 17 healthy salt-loaded men	↑Na^+^, ↓H^+^ excretion	[53]
Blood vessels	IV infusion of GLP-1 in 29 healthy subjects (2 h)	Forearm blood flow increased, ↑response to ACh challenge	[54]
	IV infusion of GLP-1 in 12 patients with T2DM and CAD (105 min)	Flow-mediated vasodilation of brachial artery doubled	[55]
	SC injection of exenatide prior to high-fat meal in 28 subjects with IGT or T2DM	Improved endothelial function after high-fat meal	[56]

ACh, acetylcholine; DBP, diastolic blood pressure; CAD, coronary artery disease; HR, heart rate; IGT, impaired glucose tolerance; Na^+^, sodium ion; SBP, systolic blood pressure; T2DM, type 2 diabetes mellitus.

Two research groups have studied the effects of GLP-1 infusion on patients with or without diabetes undergoing coronary artery bypass graft (CABG) procedures [58–59]. In one study, GLP-1 (1.5 pmol/kg/min) was administered beginning 12 h before surgery and for 48 h afterward; in the second, GLP-1 (3.6 pmol/kg/min) was administered for 12 h after transfer to the intensive care unit. Neither study observed differences in postoperative hemodynamics, but both reported reduced use of inotropic and vasoactive infusions and improved glucose control postoperatively [58–59].

The effects of GLP-1R stimulation on HF have also been investigated. In a 48-h randomized, double-blinded crossover study of 20 nondiabetic patients with compensated HF, no differences in myocardial function or metabolism were observed with administration of GLP-1 (0.7 pmol/kg/min) [60]. The results of a 3-day open-label study of GLP-1 infusion (4 pmol/kg/min) in six patients with HF and T2DM indicated that the therapy was well tolerated and there was a trend toward improved myocardial function [61]. Finally, a 5-week open-label trial of GLP-1 (2.5 pmol/kg/min infusion) in 12 patients with HF (with or without diabetes) compared with nine patients on standard therapy showed that GLP-1 therapy was associated with significant improvements in LVEF (from 21±3 to 27±3%, *P* < 0.01), maximal oxygen uptake (from 10.8 ± 0.9 mL O_2_/min/kg to 13.9 mL O_2_/min/kg, *P* < 0.001), and 6-min walking distance (from 232 ± 15 m to 286 ± 12 m, *P* < 0.001) [62]. In the latter trial, HF patients with or without diabetes appeared to benefit.

**Figure 3:**
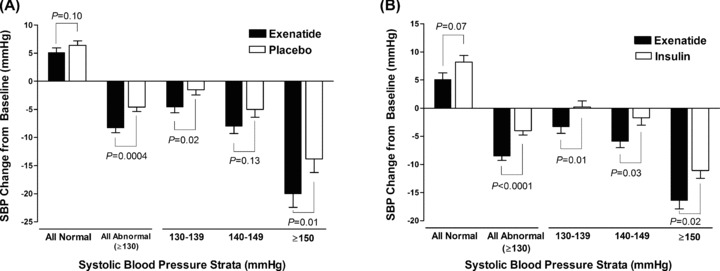
Systolic blood pressure (SBP) changes from baseline-to-endpoint (6 months) in patients with T2DM treated with exenatide (mean ± SE). (A) Exenatide versus placebo. (B) Exenatide versus insulin (Reprinted from [63]).

No phase III studies of exenatide or liraglutide have been conducted in patients with T2DM and CVD; however, blood pressure (BP) was recorded during all clinical studies. Reductions in BP were observed during studies of both exenatide and liraglutide [63–64]. Recently, 52-week follow-up data on administration of once-weekly exenatide demonstrated an systolic blood pressure (SBP) reduction of −6.2 mm Hg and a DBP reduction of −2.8 mmHg (n = 120; *P* < 0.05) [65]. A *post hoc* analysis of pooled data from six trials of exenatide bid in patients with T2DM compared with placebo and insulin (n = 2171) demonstrated a significant reduction in SBP relative to placebo and insulin comparators with a weak correlation to weight loss (*r*= 0.09, *P*= 0.002). No significant decrease in mean DBP was observed between treatment groups in this analysis. Patients with higher baseline SBP (≥150 mmHg) experienced the greatest blood pressure-lowering effects (Figure 3) [63]. A separately conducted 26-week randomized open-label trial comparing the efficacy of liraglutide qd and exenatide bid in 464 patients with diabetes demonstrated that BP decreased similarly with both treatments at 26-week (SBP) [mm Hg]: −2.51 [liraglutide] vs. −2.00 [exenatide], *P*= 0.64; DBP [mm Hg]: −1.05 [liraglutide] vs. −1.98 [exenatide], (*P*= 0.16) [49]. A meta-analysis across six trials of liraglutide (n = 1363 for 1.8 mg, n = 896 for 1.2 mg) confirmed BP reductions of ∼2.5 mm Hg with treatment and demonstrated reductions within 2 weeks of therapy, before significant weight change occurred [64]. In addition, central pulse pressure and augmentation pressure have been shown to be reduced significantly (∼8 mm Hg) by exenatide compared with insulin glargine in patients with T2DM [66].

Reductions in risk factors or markers for CVD have also been observed following treatment with GLP-1R agonists [65,67–68]. Decreases in triglycerides of 20% or more have been reported with exenatide qw or liraglutide qd treatment, but changes in other lipids have been less reproducible and may be related to weight loss [65,68]. A significant reduction in LDL-C and an increase in HDL-C (n = 151) was observed in a long-term follow-up study of exenatide bid in which patients lost −5.3 kg, but not in a 26-week study of exenatide bid versus liraglutide qd (n =∼230 per group) in which patients lost ∼3 kg [49,67]. Other surrogate markers of CVD risk reported in preliminary studies to improve during therapy with a GLP-1R agonist include C-reactive protein, adiponectin, alanine transaminase, plasminogen activator inhibitor-1, and brain natriuretic peptide [65,67–68].

In patients with T2DM, the effects of GLP-1R agonists on vasodilatation, BP, and triglycerides have been reproducible with different GLP-1R agonists studied by different researchers. As a whole, the results support additional investigation on the potential cardiovascular benefits of therapy with GLP-1R agonists. Qualitatively, the nature of the cardiovascular effects of the GLP-1R agonist class appear to differ from the cardiovascular effects of other glucose-lowering therapies, including biguanides, sulfonylureas, thiazolinediones, and insulin.

Important questions for future research include the following: Can improvements in SBP be confirmed to occur largely independently of weight loss, and what are the effects on central aortic pressure and nocturnal dipping? Is the observed tachycardia related to vasodilatation or a direct effect? Does the change in sodium excretion reduce cardiac load? Do different GLP-1R agonists have similar effects on the cardiovascular system, or are their effects meaningfully different? Can GLP-1R agonists be designed that do not increase heart rate? How do the cardiovascular effects of GLP-1R agonists compare with those of other glucose-lowering therapies or with therapies indicated to treat cardiovascular disease? Most importantly, do the observed changes in cardiovascular risk factors and markers with GLP-1R treatment translate to a reduced incidence of major adverse cardiovascular events? If so, are these effects also observed in patients without T2DM?

## The Safety and Tolerability of GLP-1R agonists

The cardiovascular safety characteristics of exenatide BID and liraglutide in clinical trial programs have been assessed, and no increase in the incidence of adverse cardiovascular events was apparent for either therapy [69][70]. In the pooled clinical trial data for exenatide BID (N = 2279 subjects), the relative risk of at least one CV event was 0.69 (95%CI 0.46–1.04) for patients treated with exenatide BID compared with the pooled comparators. However, neither the size nor duration of the clinical trials was designed to detect differences in cardiovascular outcomes. Similarly, no increase in mortality was observed in a subset of patients in the ACCORD study treated with a GLP-1 receptor agonist [71]. The only GLP-1R mechanism of action identified to date that might have a negative effect on cardiovascular safety is the slight increase in heart rate. In epidemiological studies, 20 bpm increases are associated with higher mortality over 20 years [72].

General safety issues of interest for the GLP-1R agonist class include gastrointestinal issue such as nausea and vomiting, which are usually mild to moderate and decline after the first few months of therapy. Hypoglycemia requiring assistance is rare with GLP-1R agonists except when used in combination with a sulfonylurea [1]. Potential safety concerns include an increased risk of pancreatitis, although no causal relationship has been shown; patients with T2D appear to be at higher risk for this condition [73]. Data from rat and mouse studies demonstrated an increased risk of C-cell carcinoma for liraglutide, although this has not been proven in humans [70]. Liraglutide is contraindicated in patients with a personal or family history of medullary thyroid carcinoma or multiple endocrine neoplasia syndrome type 2, and is not recommended as first-line therapy for patients inadequately controlled on diet and exercise [74]. Hypersensitivity reactions have been documented in postmarketing reports for exenatide BID, and use of exenatide BID is contraindicated in patients with severe renal impairment or end-stage renal disease or in patients with severe gastrointestinal disease (e.g., gastroparesis) [75].

## Ongoing Studies

The cardiovascular effects of GLP-1R agonists in patients with T2DM and or CVD are an active area of investigation (http://www.clinicaltrials.gov). As of October 2009, 11 studies were planned or recruiting patients, including studies on the clinical effects of exenatide or GLP-1 on outcomes of patients in the cardiac intensive care unit, post-PCI patients, post-CABG patients, and patients with HF (Table 4). These studies are essential in determining whether the unique observed effects of GLP-1R agonists on cardiovascular function depend on the model systems studied, or are a relevant clinical observation.

**Table 4:** Ongoing clinical trials related to the cardiovascular effects of GLP-1R agonists. Source: http://www.clinicaltrials.gov on October 1, 2009

Topic	Title	Outcome measures	Sponsor/reference no.
Vascular function	GLP-1 and endothelial dysfunction in metabolic syndrome	Vasodilatation capacity and glucose uptake with GLP-1 over 100 min (n = 20)	University of Rome Tor Vergata, Merck Sharp & Dohme/NCT00856700
	The effect of liraglutide on endothelial function in patients with T2DM	Forearm blood flow stimulated by ACh after 12 weeks of treatment with liraglutide, glimepiride or placebo (n = 54)	Novo Nordisk/NCT00620282
	The effect of exenatide compared to lantus insulin on vascular function in T2DM	Flow-mediated dilatation before/after a meal, nitroglycerin response, arterial stiffness. Also markers of inflammation, endothelial activation, fibrinolysis, oxidative stress (n = 72)	Joslin Diabetes Center, Amylin Pharmaceuticals Inc, Eli Lilly&Co/NCT00353834
	The vascular effects of exenatide versus metformin in patients with prediabetes	After 3 months of treatment, flow-mediated dilatation before/after a meal. Also markers of inflammation and oxidative stress (n = 50)	St Paul Heart Clinic, Amylin Pharmaceuticals Inc, Eli Lilly&Co/NCT00546728
	Effect of sitagliptin on endothelial progenitor cells	Changes in endothelial progenitor cells (involved in angiogenesis and vascular healing) after 1 month of therapy. Changes in SDF1α (n = 20)	University of Padova/NCT00968006
	Endothelial and metabolic effects of GLP-1 in coronary circulation in patients with T2DM	Coronary blood flow by arteriography 10 min after GLP-1 infusion. Coronary metabolite uptake (n = 20)	University Hospital, Gentofte, Copenhagen; Merck Sharp & Dohme/NCT00923962
Patients with CVD	IV Exenatide in coronary intensive care unit patients	IV exenatide for 24–48 h. Ave glucose value, no. of hypoglycemia measures, SAEs over the next 30 days (n = 40)	Saint Luke's Health System, Amylin Pharmaceuticals Inc, Eli Lilly&Co\NCT00736229
	Pharmacological postconditioning to reduce infarct size following primary PCI (POSTCON II)	IV exenatide or saline for 6 h in STEMI patients beginning at arrival to catheterization lab. Infarct size at 3 months by MRI, mortality at 1 month, 15 months (n = 100)	Rigshospitalet, Denmark. University Hospital, Gentofte, Copenhagen/NCT00835848
	Effects of GLP-1 on myocardial function following CABG surgery (GLP-1 CABG)	IV GLP-1 or pbo for 72 h in patients undergoing CABG. LV systolic function, hemodynamic parameters over 2 y, insulin requirements, incidence of hypo, duration and treatments in ICU (n = 48)	Johns Hopkins University/NCT00966654
	Effects of exenatide in T2DM patients with congestive heart failure	Exenatide or insulin glargine for 27 weeks. CMR to assess LVEF at −2 and 11 weeks, PET on cardiac perfusion at −2 and 26 weeks, CMR on cardiac dimensions/scarring at −2 and 26 weeks, TTE at −2 and 26 weeks. Exercise capacity at −1 and 27 weeks (n = 42)	VU University Medical Center, Amsterdam, Netherlands. Eli Lilly&Co/NCT00766857
	Evaluating use of exenatide in people with T2DM and diastolic heart failure	Exenatide versus usual care for 12 weeks. Change in aortic stiffness, changes in LV stiffness, biomarkers of glycation end products and collagen synthesis (n = 60)	National Heart, Lung, and Blood Institute/NCT00799435

ACh, acetylcholine; CABG, coronary arterial bypass graft; CMR, cardiac magnetic resonance; GLP, glucagon-like peptide; hypo, hypoglycemia; ICU, intensive care unit; LVEF, left ventricular ejection fraction; PET, Positron Emission Tomography; PCI, percutaneous coronary intervention; pbo, placebo; SAE, severe adverse event; SDF, stromal cell-derived factor; STEMI, ST-elevation myocardial infarction; T2DM, type 2 diabetes mellitus; TTE, trans-thoracic echocardiography.

The single most important issue to be addressed in this research field is the question of patients’ cardiovascular outcomes on GLP-1R agonist therapy. To provide the needed evidence, large outcome trials are planned to investigate whether liraglutide and or exenatide QW reduce adverse cardiovascular outcomes in patients with T2DM. The Exenatide Study of Cardiovascular Event Lowering (EXSCEL) trial is recruiting patients for a >5-year study of the time to first confirmed cardiovascular event in 9500 patients treated with exenatide QW or placebo (NCT01144338).

## Conclusion

Review of the existing literature supports the hypothesis that GLP-1R agonists exert physiological effects beyond glucose control that affect the cardiovascular system. While observed effects of GLP-1R agonists on triglycerides and markers of cardiovascular risk may be related to weight loss, exploratory data in both animals and humans are consistent with direct effects of GLP-1R agonists on endothelial function, excretion of sodium, improvement in SBP, limitation of ischemia/reperfusion injury, and with improved myocardial function in HF. Planned and ongoing clinical research studies will contribute significantly to knowledge of the cardiovascular effects of GLP-1R agonists. These studies may affect future patient care, as no current therapy for CVD provides the unique combination of potential actions proposed for GLP-1R agonists: improved glucose control with minimal hypoglycemia; improved endothelial function; reduced body weight, BP and serum triglycerides; improved recovery from ischemia; and improved hemodynamics in patients with reduced contractile function.
